# Синдром DICER1: клиническая гетерогенность, эндокринные проявления и особенности диагностики

**DOI:** 10.14341/probl13383

**Published:** 2023-10-16

**Authors:** Е. Э. Новокрещенных, А. А. Колодкина, О. Б. Безлепкина

**Affiliations:** Национальный медицинский исследовательский центр эндокринологии; Национальный медицинский исследовательский центр эндокринологии; Национальный медицинский исследовательский центр эндокринологии

**Keywords:** DICER1, многоузловой зоб, опухоли клеток Сертоли-Лейдига, бластома гипофиза, плевропульмональная бластома

## Abstract

Синдром DICER1 является редким наследственным заболеванием, характеризующееся прогрессирующим развитием доброкачественных и злокачественных образований преимущественно в детском и молодом возрасте. В основе данного синдрома лежит нарушение функции эндорибонуклеазы DICER, играющей значительную роль в процессинге микроРНК с последующей регуляцией контроля экспрессии онкогенов и генов-супрессоров опухолевого роста. Клиническая картина дайсеропатий весьма разнообразна и может включать как эндокринные проявления – многоузловой зоб, высокодифференцированный рак щитовидной железы, стромальные опухоли яичников, бластомы гипофиза, так и неэндокринные образования – плевропульмональную бластому, кистозную нефрому, пинеобластому, рабдомиосаркому и другие образования. Возникновение соматических мутаций гена DICER1 является результирующим этапом в патогенезе дайсеропатий, определяющим дальнейшее направление онкогенеза. В настоящее время синдром DICER1 диагностируется редко, что приводит к позднему выявлению составляющих заболевания у пациента, поздней диагностике новообразований, отсутствию семейного консультирования. Диагностика на ранних этапах заболевания, разработка программ скрининга при ведении данных пациентов позволяет минимизировать риски развития более злокачественных, агрессивных форм заболевания.

## Abstract (en)

DICER1 syndrome is a rare genetic disorder with the progressive development of malignant and non-malignant diseases in childhood. The cause of this syndrome is a dusfunction of the endoribonuclease DICER, which plays an important role in the processing of microRNAs with subsequent regulation of the control of the expression of oncogenes and tumor suppressor genes. Clinical manifestations of dyseropathies is very different and may include both endocrine manifestations – multinodular goiter, differentiated thyroid cancers, ovarian stromal tumors, pituitary blastoma, and non–endocrine formations — pleuropulmonary blastoma, cystic nephroma, pineoblastoma. The presence of somatic mutations of the DICER1 gene is a resultant stage in the pathogenesis of dyseropathies, determining the further path of oncogenesis. At present, DICER1 syndrome is diagnosed extremely rarely, which leads to late detection of the components of the disease in the patient, late diagnosis of neoplasms, lack of family counseling. Diagnosis at the early stages of the disease, the development of screening programs for the management of these patients allows minimizing the risks of developing more malignant, aggressive forms of the disease.

## ВВЕДЕНИЕ

Синдром DICER1 представляет собой редкое аутосомно-доминантное заболевание, предрасполагающее к развитию злокачественных и доброкачественных образований [1].

Изначально было доказано, что ген DICER1 играет важную роль в закладке легочной ткани в эмбриогенезе. Исследования, проведенные на мышиных моделях, доказывали, что выключение одной копии гена приводит к кистозной дилатации дыхательных путей, нарушению морфогенеза, которое напоминает раннюю стадию плевропульмональной бластомы. В 2009 г. были опубликованы данные о результатах исследования в большой группе пациентов с плевропульмональной бластомой: всем пациентам исследовался ген DICER1, выбранный как ген-кандидат, дефект которого может приводить к данному заболеванию [2]. Гетерозиготные мутации в гене DICER1 были обнаружены во всех обследованных семьях. В последующем были выявлены новые генетические ассоциации. Помимо плевропульмональной бластомы, были описаны «главные» проявления DICER1-синдрома: нефрокарцинома или кистозная нефрома, опухоли стромы полового тяжа (опухоли клеток Сертоли-Лейдига, опухоли гранулезы и арренобластомы), образования щитовидной железы, такие как многоузловой зоб, аденомы или дифференцированный рак щитовидной железы, бластомы гипофиза и другие очень редко встречающиеся состояния — медуллоэпителиома, эмбриональная рабдомиосаркома шейки матки, хондромезенхимальная носовая гамартома, почечная саркома, пинеобластома и единичные описания опухоли Вильмса, семиномы и рака печени. Заболевание характеризуется ранним началом, до 40 лет, но чаще — в детском или подростковом возрасте.

Предполагаемая распространенность патогенных вариантов DICER1 в общей популяции составляет 1:10600, и из-за гетерогенных клинических особенностей и редкости этого синдрома его диагностика остается проблемой для клиницистов [3]. В онкологической популяции распространенность этого заболевания оценивается как 1:4600 [4][5].

Первые симптомы появляются в первые два десятилетия жизни; существенных различий по полу или этническим группам нет [3][6].

Ген DICER1 расположен на хромосоме 14q32.13 и кодирует белок массой около 200 кДа — эндорибонуклеазу семейства РНКаз III, участвующую в процессе производства и созревания большинства микроРНК (миРНК). МикроРНК (миРНК) являются небольшими некодирующими последовательностями РНК размером ~22 нуклеотида и, как известно, играют ключевую роль в посттранскрипционной регуляции матричной РНК (мРНК) [5][7]. Являясь цитоплазматическими регуляторами экспрессии генов, микроРНК осуществляют посттранскрипционную регуляцию матричной РНК (мРНК) посредством ее деградации или трансляционной репрессии.

Предшественник микроРНК, первичная микроРНК (премиРНК), представляет собой последовательность размеров ~200 нуклеотидов, организованных в структуры «шпильки» из 60 нуклеотидов. Под воздействием нуклеарного фермента РНКазы III Drosha происходит отщепление «шпильки» с образованием предшественника микроРНК пре-микроРНК (премиРНК) с дальнейшей транспортировкой в цитоплазму с помощью экспортина 5 [8].

Цитоплазматический аналог фермента Drosha, РНКаза III Dicer осуществляет процессинг премиРНК с образованием дуплексов микроРНК (д-миРНК), состоящих из 18–22 нуклеотидов. В дальнейшем дуплекс микроРНК участвует в создании РНК-индуцированного комплекса выключения белка (RISK, RNA-induced silencing complex), представляющего собой сложную структуру, которая состоит из белков семейства Argonaute (AGO), РНК-связывающего белка трансактивационного ответа (TARBP2) и РНКазы III Dicer [9]. Данный комплекс, взаимодействуя с некодирующим участком 3’-конца матричной РНК (мРНК), приводит к предотвращению ее трансляции или полной деградации молекулы [10] (рис. 1).

**Figure:**
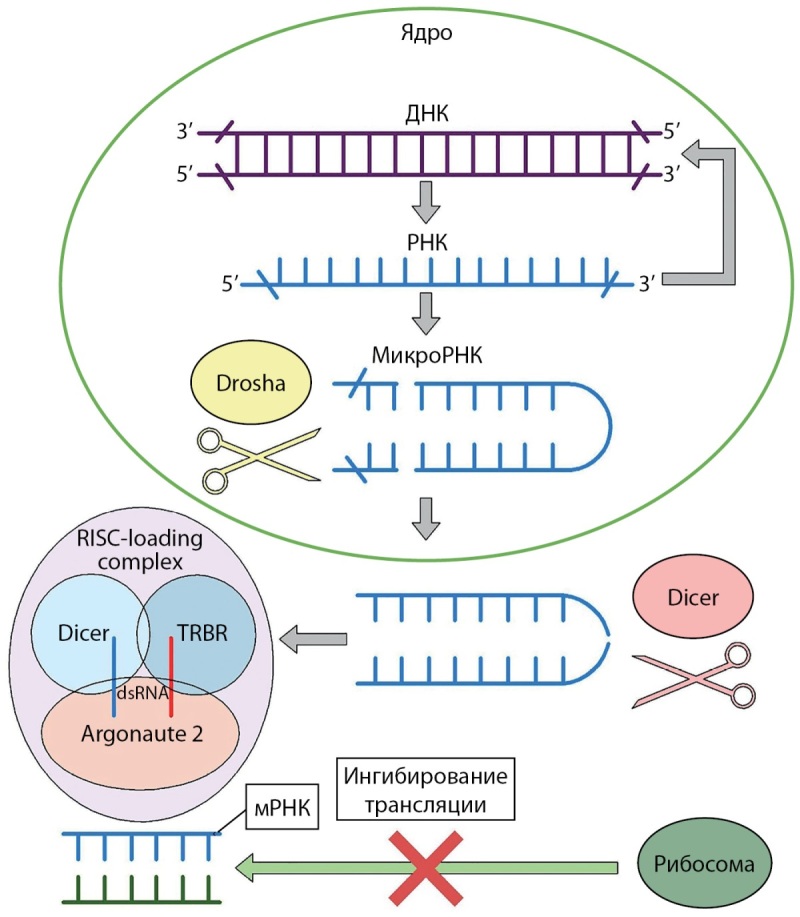
Рисунок 1. Механизм действия эндорибонуклеазы DICER1. Figure 1. Mechanism of action of endoribonuclease DICER1.

Таким образом, дерегуляция микроРНК имеет проонкогенный эффект: сверхэкспрессия одной микроРНК может ингибировать трансляцию белка гена — супрессора опухоли, в то время как подавление другой микроРНК может повышать уровень белка-онкогена [11–13]. В целом контроль экспрессии генов, опосредованных микроРНК, имеет решающее значение для реакции клеток на окислительный стресс, гипоксию и повреждение ДНК.

## КЛИНИЧЕСКИЕ ПРОЯВЛЕНИЯ СИНДРОМА DICER1

Клиническая картина дайсеропатий весьма вариабельна и может включать как эндокринные проявления — многоузловая гиперплазия щитовидной железы, стромальные опухоли яичников, бластома гипофиза, так и неэндокринные образования — плевролегочная бластома, кистозная нефрома, пинеобластома. Данные новообразования могут протекать как изолированно, так и в сочетании друг с другом.

## Эндокринные проявления дайсеропатий

1. Многоузловой зоб

Многоузловой зоб, ассоциируемый с синдромом DICER1, является наиболее часто встречающимся компонентом данного синдрома. Быстропрогрессирующий рост узловых образований и высокий риск развития дифференцированного рака щитовидной железы у детей раннего возраста требует комплексного подхода и персонализированной тактики ведения данных пациентов.

По данным Khan et al., патологические изменения щитовидной железы были выявлены у 75% женщин и 17% мужчин с установленным синдромом DICER1 [14]. В структуре тиреопатий при синдроме DICER1 преобладает многоузловой зоб различной степени пролиферации, фолликулярные аденомы, реже встречается высокодифференцированный рак щитовидной железы — папиллярная и фолликулярная карциномы. Также сообщалось о случае выявления низкодифференцированной карциномы щитовидной железы в составе синдрома DICER1 [15].

До недавнего времени считалось, что при синдроме DICER1 вероятность развития злокачественных образований в составе многоузловой гиперплазии щитовидной железы весьма небольшая и соответствует выявлению высокодифференцированного рака щитовидной железы в общей популяции. Однако, по данным исследований, проведенных за последнее десятилетие, высокодифференцированный рак щитовидной железы при мутациях в гене DICER1 встречается в 16–18 раз чаще, чем в общей популяции [14]. Стоит отметить, что злокачественные поражения щитовидной железы при дайсеропатиях как правило имеют малую вероятность метастазирования в лимфатические узлы и относятся к клинической группе низкого риска возможного рецидива [16].

Патологические изменения щитовидной железы при синдроме DICER имеют характерную эхографическую картину с преобладанием в структуре тиреоидной ткани множественных образований (более трех) смешанной кистозно-солидной структуры, а также изоэхогенных солидных узлов. Для пациентов старшего возраста характерно наличие микро- и макрокальцинатов в структуре узловых образований [17].

Проведение тонкоигольной аспирационной биопсии является важным диагностическим методом для определения дальнейшей тактики ведения данных пациентов, однако ее возможности ограничены трудностями в осуществлении пункции абсолютно всех образований, учитывая многоузловой характер поражения. Как правило, в детской практике осуществляется биопсия 2–3 наиболее подозрительных образований, что не позволяет создать полноценную картину о цитологическом строении всей пораженной железы.

При цитологическом подтверждении наличия высокодифференцированного рака щитовидной железы, фолликулярной аденомы или же при большом объеме зоба, вызывающего компрессию трахеи и/или имеющего выраженный косметический дефект, методом выбора является хирургическое лечение. Учитывая тенденцию к быстрому росту узловых образований с высокой вероятностью развития злокачественного поражения, предпочтительнее проведение тиреоидэктомии. По данным литературы, пациентам с гемитиреоидэктомией по поводу многоузлового зоба в составе синдрома DICER1 в течение короткого периода требовалась повторная операция в объеме окончательной тиреоидэктомии в связи с прогрессирующим ростом узловых образований оставшейся доли щитовидной железы [14].

2. Опухоли стромы полового тяжа

Опухоли стромы полового тяжа относятся к злокачественным новообразованиям яичников, содержат клетки зернистой оболочки фолликулов, клетки внутренней соединительнотканной оболочки (тека-клетки), клетки Сертоли и Лейдига, фибробласты стромального происхождения. В составе синдрома чаще всего наблюдаются опухоли клеток Сертоли-Лейдига, реже — гранулезоклеточные опухоли ювенильного типа и арренобластомы. Опухоли могут образовываться как в одном, так и в обоих яичниках, а между возникновением опухолей может проходить несколько лет.

Опухоли клеток Сертоли-Лейдига являются достаточно редкими среди всех злокачественных образований яичников (менее 1%) [18], однако данные многочисленных исследований сообщают, что более 60% пациентов с Сертоли-Лейдига клеточными образованиями имели мутацию в гене DICER1 [19, 20]. По данным de Kock et al., были изучены 34 пациента с подобными образованиями, и в 88% выявлены патологические изменения в гене DICER1 [21].

Клиническая картина очень схожа с другими новообразованиями яичников; пациентки отмечают вздутие, боли в нижних отделах живота. В ряде случаев возникают признаки вирилизации за счет гиперпродукции андрогенов, такие как гирсутизм, барифония, появление большого количества акне, гипертрофия клитора. Данный вид новообразований в составе DICER1-синдрома развивается преимущественно в молодом возрасте, 75% установленных диагнозов приходится на диапазон от 12 до 25 лет.

Тактика лечения зависит от стадии заболевания, состояния капсулы, степени дифференцировки опухоли. При IА стадии заболевания стараются ограничиться односторонним удалением придатков матки. При недифференцированных опухолях необходимо проводить послеоперационную химиотерапию [20].

Ювенильные гранулезоклеточные опухоли могут быть различных размеров — от практически неопределяемых до заполняющих всю брюшную полость, часто кистозносолидного строения; причем кисты встречаются как одиночные, так и множественные, отмечаются очаги некроза и кровоизлияний. Клетки опухоли богаты цитоплазмой, ядра с неровной поверхностью и выраженной митотической активностью.

Клинически пациенты жалуются на боли в области живота, поясницы, чувство сдавления соседних органов. В случае возникновения гормональноактивных опухолей прослеживаются симптомы, обусловленные влиянием эстрогенов или андрогенов. В детском возрасте могут проявляться признаки преждевременного полового развития. В ряде случаев может наблюдаться картина «острого живота» из-за нарушения целостности капсулы опухоли и внутреннего кровотечения. Для диагностики используют определение уровней β-ХГЧ, АФП, ЛДГ, ингибина B. Для ингибина В доказана высокая специфичность. Заболевание чаще протекает благоприятно [21].

Арренобластома — это чрезвычайно редкая опухоль, возникающая из недифференцированной мезенхимы, что обусловливает ее бисексуальную потенцию. В связи с андрогенной стимуляцией у больных преобладают признаки вирилизации. В большинстве случаев опухоль доброкачественная, однако может быть и злокачественное течение. В связи с дефектами DICER1 описано 2 пациента [22]. Опухоли обычно диагностируются на ранней стадии и требуют только хирургического лечения [22].

3. Бластома гипофиза

Бластома гипофиза — очень редкая и агрессивная, по-видимому, врожденная гипофизарная опухоль, одно из тяжелых проявлений DICER1-синдрома. В настоящее время это единственное заболевание, при котором объемное образование гипофиза обозначается термином «бластома», к данному гистологическому типу она отнесена потому, что имеет признаки эмбриональной ткани, в ней присутствует эпителий и железистая ткань, характерные для кармана Ратке, структурно напоминает аденогипофиз 10-12-й недели эмбриогенеза, когда выражена дифференцировка только кортикотрофов и соматотрофов.

В настоящее время в литературе описано 14 клинических случаев гипофизарной бластомы. Для всех пациентов был характерен ранний дебют заболевания — от 7 до 18 месяцев, что позволяет расценивать образование как врожденное [23]. Заболевание у всех пациентов проявлялось болезнью Кушинга, повышением уровня АКТГ и кортизола, офтальмоплегией, у одной пациентки отмечалась еще и клиника несахарного диабета [23]. В 40% случаев гипофизарная бластома оказалась летальной. Подробного описания причины летальности в литературе не указано, смерть пациентов наступала из-за локальных эффектов, опухоли вызывали повышенное внутричерепное давление, нарушение ликвородинамики и повреждение окружающих тканей, осложнений гиперкортизолизма. Отмечается склонность к рецидивированию заболевания [24].

Гипофизарная бластома — чрезвычайно редкая опухоль, патогномоничная для DICER1-синдрома. При отсутствии возможности провести молекулярно-генетическое исследование таких пациентов надо обследовать и наблюдать как пациентов с синдромом предрасположенности к опухолям, обусловленными дефектом DICER1.

## Неэндокринные проявления дайсеропатий

1. Плевропульмональная бластома

Плевропульмональная бластома является наиболее распространенным неэндокринным проявлением синдрома DICER1 с прогрессирующим развитием в течение первых 4–5 лет жизни. В 2009 г. Hill et al. сообщили о первом описании мутации в гене DICER1, выявленной при семейной форме плевропульмональной бластомы [2]. По данным международного реестра плевропульмональных бластом, в 66% случаев были выявлены различные гетерозиготные варианты в гене DICER1 [25].

Выделяют несколько вариантов плевропульмональных бластом, представляющих собой стадийное течение заболевания с постепенной прогрессией от доброкачественных кистозных в злокачественные солидные формы. I тип характеризуется множественным кистозным новообразованием, обладающим злокачественным потенциалом-трасформацией в следующие варианты плевропульмональных бластом. Ir тип (регрессирующий) также представляет собой кистозные разрастания, однако не содержит злокачественные клетки. II тип характеризуется сочетанием кистозных и солидных структур, а III тип состоит преимущественно из солидных злокачественных клеток. При II и III типах отмечается более агрессивный характер течения с большой вероятностью метастазирования преимущественно в головной мозг [26][27].

Детям из групп риска рекомендовано проведение рентгенографии грудной клетки на первом году жизни с целью выявления возможных кистозных поражений легких. У пациентов с доказанной мутацией в гене DICER1 необходимо проведение мультиспиральной компьютерной томографии легких до 9 месяцев жизни. В случае отсутствие визуализации патологии рекомендовано повторное исследование в возрасте 2,5 года, что предшествует пику заболеваемости плевропульмональных бластом II и III типов.

Кистозные новообразования при I и Ir (регрессирующем) типе требуют хирургического вмешательства и являются прогностически благоприятными — 5-летняя выживаемость составляет 91%. Кистозно-солидные и солидные бластомы при II и III типах подлежат как хирургическому, так и химиотерапевтическому лечению и имеют худший прогноз — 5-летняя выживаемость составляет 71 и 53% соответственно [28]. Раннее выявление плевропульмональных бластом позволяет провести своевременное радикальное хирургическое лечение и не допустить трансформацию в более злокачественные формы.

2. Кистозная нефрома

Кистозная нефрома представляет собой одностороннее доброкачественное образование почки, возникающее преимущественно в детском возрасте до 4 лет. Данная опухоль может быть представлена множественными кистозными разрастаниями, по своему строению напоминающими плевропульмональную бластому I типа, что подтверждает единый механизм развития образований [29]. По данным литературы, частота встречаемости патогенных вариантов гена DICER1 у пациентов с кистозными нефромами составляет около 70% [20].

Клинически кистозная нефрома проявляется увеличением объема живота вследствие разрастания объемного образования, также возможно появление болезненности в животе, гематурии. В некоторых случаях сообщалось о трансформации кистозной нефромы в анапластическую саркому почки [30][31]. Хирургическое удаление отдельных кист в большинстве случаев не является эффективным вследствие большой вероятности рецидива [32]. Методом выбора хирургической тактики является проведение радикальной нефрэктомии.

3. Пинеобластома

Пинеобластома представляет собой высокоагрессивную злокачественную опухоль шишковидной железы головного мозга. Данное образование относится к примитивным нейроэктодермальным образованиям, частота встречаемости — менее 1% среди всех опухолей головного мозга [33]. Пинеобластомы обладают инфильтративным ростом, разрушая ткань среднего мозга, часто прорастают в мягкую мозговую оболочку с распространением в субарахноидальное пространство. Клинические проявления связаны с общемозговой симптоматикой — головная боль, головокружение, тошнота, рвота, а также быстрым присоединением гидроцефалии. Прогноз крайне неблагоприятный, учитывая высокую скорость метастазирования, выживаемость после выявления пинеобластомы не превышает 1 год. Помимо соматических мутаций DICER1, при пинеобластомах также были выявлены мутации в гене DROSHA, также связанных с нарушением функционирования микроРНК [34].

4. Назальная хондромезенхимальная гамартома

Назальная хондромезенхимальная гамартома является редкой доброкачественной опухолью синоназального тракта у детей. По данным литературы, зарегистрировано около 48 случаев, из них 18 у детей младше одного года. Средний возраст выявления — 9,6 года, также описано 6 случаев диагностики во взрослом возрасте [35].

Гамартома имеет смешанное морфологическое строение, состоит преимущественно из мезенхимального и хрящевого компонентов. Клинические проявления обусловлены вариантом локализации опухоли в полости носа или околоносовых пазухах и наличием компрессии близлежащих структур. Симптомы варьируются от заложенности носа и хронических синуситов до нарушения зрения, невралгии, головных болей.

По данным Mason et al., из 47 описанных ранее пациентов с хондромезенхимальной назальной гамартомой у 11 также была диагностирована плевропульмональная бластома, а у 5 из них – опухоли клеток Сертоли-Лейдига. Также у 1 пациента был выявлен многоузловой зоб, а другой наблюдался по поводу папиллярной карциномы щитовидной железы [35]. В 2013 г. описан единственный случай трансформации хондромезенхимальной назальной гамартомы в злокачественную форму [36]. Методом терапии является проведение полной хирургической резекции образования с целью предупреждения возможного рецидива.

По данным Stewart et al., у 6 из 8 обследованных пациентов с сочетанием плевропульмональной бластомы и назальной хондромезенхимальной гамартомы были выявлены мутации в гене DICER1, что позволяет рассматривать данное образование как часть клинического спектра дайсеропатий [37]. Учитывая выявление гамартомы преимущественно у детей раннего возраста, данным пациентам необходимо проведение дальнейшего наблюдения и скрининга всех возможных компонентов синдрома DICER1.

5. Медуллоэпителиома цилиарного тела

Медуллоэпителиома представляет собой редкую врожденную эмбриональную опухоль, образующуюся из клеток эпителия цилиарного тела. Данное образование может встречаться как в педиатрической, так и во взрослой когорте пациентов, однако преимущественно выявляется на первом десятилетии жизни [38]. Учитывая низкую частоту встречаемости, отмечаются сложности в дифференциальной диагностике с другими внутриглазными образованиями, такими как ретинобластома, шваннома, а также воспалительная гранулема [39]. У большинства пациентов отмечается снижение остроты зрения, боль, лейкокория или образование в передней камере глаза. Также в дальнейшем возможно присоединение вторичных признаков — катаракты и глаукомы, что чаще всего и является причиной обращения к специалистам [40].

Медуллоэпителиома циллирного тела может иметь как доброкачественный, так и злокачественный характер течения, и имеет тенденцию к метастазированию при распространении за пределы глазного яблока. Методами лечения при небольших размерах образования является криотерапия, хирургическая резекция, а также лучевая терапия. При больших распространенных опухолях, а также рецидивирующих образованиях методом выбора является энуклеация пораженного глаза [40].

По данным реестра плевропульмональных бластом [25], у 4 детей с патогенной мутацией гена DICER1 диагностирована медуллоэпителиома цилиарного тела в возрасте 4, 6, 8 и 9 лет.

## Молекулярные механизмы развития дайсеропатий

С момента первого описания патологических вариантов в гене DICER1 в 2009 г. долгое время считалось, что наличие одной измененной копии гена достаточно для предрасположенности организма к избыточному опухолевому росту. Однако, по данным последних исследований, большое внимание уделяется описанию соматических мутаций гена DICER1, обнаруженных непосредственно в опухолевых тканях [33][41].

В 1971 г. Кнудсоном была предложена так называемая «Гипотеза о двух попаданиях» [42], согласно которой предполагается, что для возникновения патологического процесса в организме необходимо наличие двух различных мутаций в каждой аллели, и что только одной мутации в единственной аллели недостаточно, чтобы вызвать образование опухоли. Наследуемая герминативная мутация как правило относительно безвредна, однако в тандеме со второй соматической мутацией может приводить к возникновению онкологического процесса. Таким образом, гипотеза двух попаданий характеризует механизм, с помощью которого происходит деактивация гена-супрессора опухолевого роста [43].

Данная теория применима и к мутациям гена DICER1, и роли эндорибонуклазы Dicer как белка-онкосупрессора. Недавние исследования показали, что пациенты с синдромом DICER1 не только унаследовали патологические изменения в одной аллели гена DICER1, но также приобрели соматическую мутацию во второй аллели [22]. Безусловно, герминативные мутации гена DICER1 предрасполагают организм к повышенному риску развития как доброкачественных, так и злокачественных опухолей. Однако возникновение в ходе онкогенеза второй соматической мутации в другой аллели в тандеме с наследуемой мутацией может приводить к более агрессивным редким видам рака.

Данные соматические мутации были обнаружены в домене РНКазы IIIb гена DICER1, которые являются генетическими «горячими точками» для возникновения изменений нуклеотидных последовательностей [41][44]. Эти мутации влияют на способность домена связывать ионы металлов, нарушая тем самым каталитический сайт фермента и, таким образом, снижая продукцию микроРНК.

По данным исследования Seki et al., из 11 пациентов с диагностированными плевропульмональными бластомами, ассоциированными с мутацией гена DICER1, у 8 также были выявлены мутации домена РНКазы IIIb DICER1 [45].

При описании 10 пациенток с опухолями из клеток Сертоли-Лейдига в составе синдрома DICER1 у 6 из 10 (60%) были выявлены соматические мутации «горячих точек» гена DICER1 [46]. Наличие соматических мутаций характеризовалось клиническими проявлениями гиперандрогенемии, вторичной аменореей, вирилизацией, тогда как у пациенток, имеющих только герминативную мутацию, данные симптомы отсутствовали.

Таким образом, возникновение соматических мутаций гена DICER1 является результирующим этапом в патогенезе дайсеропатий, определяющим дальнейшее направление онкогенеза. Приобретение мутаций «горячих точек» домена РНКазы IIIb в процессе эмбриогенеза ведет к раннему развитию заболевания с обширным мультиорганным поражением [6].

## Показания для молекулярно-генетической диагностики синдрома DICER1

Молекулярно-генетическую диагностику синдрома DICER1 рекомендовано проводить в случае наличия одного большого критерия или же двух и более второстепенных критериев (таблица 1).

**Table:** Таблица 1. Критерии диагностики синдрома DICER1 Table 1. Diagnosis criteria for DICER1 syndrome

«Большие» критерии	«Малые» критерии
•Плевролегочные бластомы •Кисты легких в детском возрасте (множественные или двусторонние) •Грудная эмбриональная рабдомиосаркома •Кистозная нефрома •Саркомы мочевыделительного тракта •Сертоли-Лейдига клеточная опухоль яичников •Гинандробластома •Рабдомиосаркома тела или шейки матки •Многоузловой зоб или рак щитовидной железы у двух или более родственников первой степени родства или семейный анамнез синдрома DICER1 •Детский возраст возникновения многоузлового зоба или дифференцированного рака щитовидной железы •Носовая хондромезенхимальная гамартома •Пинеобластома •Бластома гипофиза	•Киста(кисты) легкого у взрослых •Ренальная киста (кисты) •Опухоль Вильмса •Многоузловой зоб или дифференцированный рак щитовидной железы у взрослого •Рабдомиосаркома (не легких и не органов малого таза) •Низкодифференцированные нейроэндокринные опухоли •Макроцефалия

## ЗАКЛЮЧЕНИЕ

За более чем десятилетнюю историю изучения синдрома DICER1 его проявления становятся все более многогранными и распространенными. Широкий спектр клинических особенностей требует многопрофильного подхода и персонализируемой тактики ведения пациентов. Молекулярно-генетическое исследование гена DICER1 в педиатрической практике необходимо проводить в случае сочетания двух или более признаков дайсеропатий, а также при диагностике многоузловой быстропрогрессирующей гиперплазии щитовидной железы в детском и молодом возрасте, и при наличии отягощенного наследственного анамнеза — выявлении у родственников первой степени родства объемных образований различных локализаций (щитовидная железа, легкие, яичники, почки) преимущественно в раннем возрасте. Ранняя диагностика и планирование эффективного скрининга всех возможных проявлений дайсеропатий позволяет избежать трансформации в злокачественные формы заболевания.

## ДОПОЛНИТЕЛЬНАЯ ИНФОРМАЦИЯ

Источники финансирования. Работа проведена в рамках темы госзадания 123021000039-3 «Молекулярно-генетические маркеры стратификации риска прогрессирования/рецидива рака щитовидной железы».

Конфликт интересов. Авторы декларируют отсутствие конфликта интересов.

Участие авторов. Новокрещенных Е.Э., Колодкина А.А. — поисково-аналитическая работа и подготовка финальной версии статьи; Безлепкина О.Б. — редактирование текста, внесение ценных замечаний. Все авторы одобрили финальную версию статьи перед публикацией, выразили согласие нести ответственность за все аспекты работы, подразумевающую надлежащее изучение и решение вопросов, связанных с точностью или добросовестностью любой части работы.
